# A retrospective metatranscriptomic study of respiratory pathogens causing adult community-acquired pneumonia in Wuxi, China, before the pandemic

**DOI:** 10.7717/peerj.20774

**Published:** 2026-02-10

**Authors:** Yan-Jun Kang, Juan Liu, Yumeng Gao, Yujun Chen, Yan Wang, Chao Shi, Yuan Shen

**Affiliations:** 1 Affiliated Children’s Hospital of Jiangnan University, Wuxi, China; 2 Wuxi Center for Disease Control and Prevention, Wuxi, China; 3 Nanjing Medical University, Nanjing, Jiangsu, China

**Keywords:** Community-acquired pneumonia (CAP), Metatranscriptomic sequencing, Respiratory pathogens, Microbial diversity, Pre-pandemic baseline

## Abstract

The COVID-19 pandemic has substantially altered respiratory pathogen circulation, underscoring the critical need for pre-pandemic baseline data to interpret current epidemiological trends. To establish this baseline, we employed metatranscriptomic sequencing to characterize the etiology of community-acquired pneumonia (CAP) in 20 adult patients hospitalized in Wuxi, China, during 2018–2019. Following ribosomal RNA depletion, sequencing data were analyzed using a stringent dual-filter strategy (RPM ≥ 100 and Z-score ≥ 2) to identify high-confidence pathogens. Our analysis revealed a complex, polymicrobial landscape. Bacterial pathogens predominated, with *Streptococcus* species detected in 25% of cases. The frequent co-occurrence of oral anaerobes (*e.g.*, *Prevotella*, *Veillonella*, *Rothia*) suggested that aspiration-driven polymicrobial infections were a key pathogenic mechanism. Viral pathogens were also prominent, with *Orthorubulavirus hominis* and Human respirovirus 1 showing significant transcriptional activity. Notably, our approach enabled the discovery and characterization of two divergent viral strains: a novel Rhinovirus B strain (AP81) with only 90.52% nucleotide identity to its closest relative, and a picobirnavirus phylogenetically distinct from human strains (94.90% identity to a simian-derived virus). Fungal detection was minimal, with only *Candida albicans* meeting the criteria in a single case. In conclusion, this study provides a crucial pre-pandemic baseline of CAP etiology in Wuxi. It highlights the power of metatranscriptomics to not only define common etiological agents but also to uncover novel viral diversity and reveal the polymicrobial complexity of respiratory infections, offering critical insights for future surveillance and clinical management.

## Introduction

Community-acquired pneumonia (CAP) remains a significant public health concern in China, with incidence rates higher than those reported in Western countries (Zhu et al., 2018). Although CAP disproportionately affects children, it remains a significant cause of mortality among adults, particularly the elderly and those with underlying chronic conditions (Gonçalves-Pereira et al., 2025). In China, the incidence of adult CAP ranges from 29.8 to 221.0 cases per 10,000 individuals, which is considerably higher than the 24.8 to 26.7 cases per 10,000 adults reported in the United States. Notably, patients aged 65 and above account for 37.2% ± 7.9% of all CAP cases in China and experience the highest mortality rate (Zhu et al., 2018).

The etiology of CAP is highly diverse, encompassing a wide range of viruses, bacteria, fungi, and other microorganisms, with concurrent infections by multiple pathogens being common (Liu et al., 2023a). Given the geographical variability in pneumonia etiology, diagnostic and treatment strategies must be tailored to the locally prevalent pathogens. However, conventional diagnostic methods fail to identify causative pathogens in approximately half of adult CAP patients in China, highlighting significant limitations in current diagnostic approaches (Musher & Thorner, 2014). Metatranscriptomic profiling, which utilizes RNA sequencing, has emerged as a crucial solution to these challenges, enabling the simultaneous detection of various pathogen types and facilitating the discovery of novel or previously uncharacterized pathogens (Shi et al., 2022). The comprehensive and sensitivity nature of this method can provide more accurate information for clinical diagnosis.

The COVID-19 pandemic and associated non-pharmaceutical interventions (NPIs) have profoundly disrupted the existing epidemiological landscape of respiratory pathogens (Principi et al., 2023). Following the relaxation of these measures in late 2022 and throughout 2023, numerous countries reported sharp, out-of-season surges in respiratory infections, particularly influenza and RSV, often with altered age distributions and severity patterns (Wu et al., 2025; Yue et al., 2025; Zhao et al., 2024). This phenomenon has been attributed to accumulated susceptibility in populations with waned immunity due to reduced pathogen exposure during the pandemic, colloquially termed “immunity debt” (Munro & House, 2024). These post-pandemic shifts have altered pathogen prevalence, disrupted traditional seasonal patterns, and potentially changed the competitive dynamics among co-circulating respiratory pathogens.

Wuxi, a major city in Jiangsu Province, Eastern China, has a population of over 7.5 million and holds significant economic importance within the Yangtze River Delta region. Jiangsu Province exhibits a standardized incidence rate of CAP at 12.06 (95% CI [10.12–14.01]) per 1,000 person-years (Sun et al., 2020), underscoring the urgent need for improved surveillance and targeted interventions. Despite this recognized disease burden, there remains a notable lack of comprehensive epidemiological data on adult CAP specifically in Wuxi. While regional studies from the Yangtze River Delta prior to the COVID-19 pandemic revealed a complex and diverse etiology characterized by a mix of viral and bacterial pathogens—with human rhinovirus (HRV), influenza A virus, *Streptococcus pneumoniae*, and *Mycoplasma pneumoniae* being the most frequently identified agents (Chen et al., 2015; Wang et al., 2024; Xu et al., 2019)—detailed baseline data using unbiased molecular methods remain scarce.

To address this knowledge gap and establish pre-pandemic baseline data, we utilized metatranscriptomics to investigate the etiological spectrum in 20 adult pneumonia cases in Wuxi from 2018–2019. This study aimed to generate valuable baseline data on regional CAP etiologies, providing a critical reference point for understanding post-pandemic changes and informing future public health surveillance and clinical practice in the region.

## Materials and Methods

### Patient enrollment and sample collection

This retrospective study employs metatranscriptomic sequencing to systematically characterize the pathogen spectrum among adult pneumonia patients in Wuxi, China, during the pre-COVID-19 pandemic period (2018–2019). From 2018 to 2019, we recruited adult inpatients diagnosed with CAP from several hospitals in Wuxi, China. Inclusion criteria were patients aged 18 years and older with a clinical and radiological diagnosis of CAP, as defined by the diagnosis and treatment of community-acquired pneumonia in adults: 2016 clinical practice guidelines by the Chinese Thoracic Society. Key exclusion criteria, based on the scope of the guidelines, were hospital-acquired pneumonia and severe immunosuppressive conditions (Cao et al., 2018). Additionally, for the specific purposes of this study, patients who had received antibiotics within 48 h prior to admission or were unable to provide a sputum sample of adequate quality were also excluded. The detailed inclusion and exclusion criteria are provided in Supplementary Material 1. Written informed consent was obtained from all participants included in the study. A total of 20 patients meeting these criteria were enrolled in this study. This study received approval from the Ethics Committee of Wuxi Center for Disease Control and Prevention (Approval No. 2023-04).

Deep sputum samples were collected from all patients on the day of admission, ensuring minimal contamination with oral flora. Patients were instructed to rinse their mouths with sterile water before expectorating sputum directly into sterile containers. After collection, the sputum samples were immediately preserved in RNAlater (Qiagen, Hilden, Germany) protective solution and rapidly frozen at −80 °C until further processing. All samples were handled with strict aseptic techniques to prevent cross-contamination.

### Metatranscriptomic sequencing and data processing

Total RNA was extracted from 300 µL sputum samples using the RNeasy Plus Universal Kit (Qiagen) according to the manufacturer’s instructions. RNA quantity and quality were assessed using the Agilent 2100 Bioanalyzer (Agilent Technologies, Santa Clara, CA, USA). Subsequently, Host ribosomal RNA (rRNA) was depleted using the Trio RNA-Seq Kit (NuGEN Technologies, San Carlos, CA, USA), which also facilitated the construction of Illumina-compatible sequencing libraries. The libraries underwent 150 bp paired-end sequencing on an Illumina HiSeq X-Ten platform at BGI Genomics.

Bioinformatic analysis of the raw sequencing data involved adaptor trimming and filtering of non-complex and duplicated reads using BBmap (Bushnell, 2014). The remaining reads were mapped to the human reference genome (GRCh38/hg38) and SILVA rRNA databases to remove human and ribosomal RNA sequences. The resulting non-host reads were processed through a pathogen discovery pipeline as described by Shi et al. (2022).

### Microbial identification, quantification, and pathogen filtering

To accurately identify microbial agents and systematically distinguish them from background commensals and potential contaminants, we implemented a robust bioinformatic pipeline combining read-based classification with a stringent statistical filtering strategy.

Our primary method for taxonomic classification and quantification utilized Kraken2 (v2.1.2) with a standard database built from NCBI’s RefSeq libraries for bacteria, archaea, viruses, and fungi. The abundance of each classified taxon was then estimated at the species level using Bracken (v2.8), which generated reads per million (RPM) values for each microbe within a sample (Lu et al., 2022). This read-based approach is highly effective for accurately quantifying known microbes and is less susceptible to artifacts that can arise from sequence assembly, thus providing a more reliable foundation for bacterial and fungal analysis.

In parallel, to enable the discovery of potentially novel or highly divergent viruses not well-represented in standard databases, a supplementary assembly-based pipeline was employed. Raw reads were assembled into contigs using MEGAHIT (v1.2.9) (Li et al., 2015). These contigs were then queried against the NCBI non-redundant protein (nr) database using DIAMOND blastx (v2.1.1) (Buchfink, Reuter & Drost, 2021) to identify viral sequences.

All microbes identified through either pipeline were then subjected to a stringent, two-dimensional filtering strategy inspired by the framework of Zinter et al. (2019) to isolate the most likely etiological agents. This strategy evaluates each microbe based on two criteria:

Intra-sample abundance: RPM, representing the microbe’s prevalence within a single sample.

Inter-sample uniqueness: Quantified with a Z-score of the log_10_-transformed RPM values, indicating how many standard deviations a microbe’s abundance in one sample is from its mean abundance across the entire cohort. The Z-score was calculated as:

\begin{eqnarray*}\mathrm{Z}=(\mathrm{x}-\mu )/\sigma \end{eqnarray*}

where *x* is the log_10_(RPM) of a microbe in the sample of interest, *μ* is the mean of its log_10_(RPM) across all samples, and *σ* is the standard deviation.

A microbe was identified as a significant potential pathogen only if it met a unified set of criteria in a given sample: RPM ≥ 100 and Z-score ≥ 2. This data-driven filter was applied uniformly to bacteria, fungi, and viruses and proved critical for increasing the signal-to-noise ratio by removing ubiquitous, low-abundance organisms often associated with contamination or the stable commensal microbiome.

For significant viral species that passed this filter, further validation was performed. Reads were mapped back to the assembled viral contigs using Bowtie2 (v2.4.2) to confirm genome coverage (Langmead & Salzberg, 2012). Finally, all microbes passing these filters were cross-referenced with the Catalogue of Pathogenic Microorganisms of Human Infectious Diseases to confirm their clinical relevance (National Health Commission of the People’s Republic of China, 2023).

### Phylogenetic analysis

For significant viral species identified through the filtering strategy, phylogenetic analysis was performed to characterize strain relationships. Reference sequences were selected using a two-step approach: (1) BLASTn and BLASTx searches identified the most closely related sequences to our assembled viral contigs (top hits with ≥80% identity were selected); and (2) representative strains from major known genotypes or subtypes were included to provide broader phylogenetic context.

These selected reference sequences, along with our assembled contigs, were aligned using MAFFT (v7.490). Ambiguously aligned regions were subsequently removed using Trimal (v1.2). Finally, phylogenetic trees were constructed using the maximum likelihood approach in PhyML (v3.0) with the General Time Reversible (GTR) substitution model and 1,000 bootstrap replicates to assess branch support.

## Results

### Patient demographics and sequencing data

**Figure 1:**
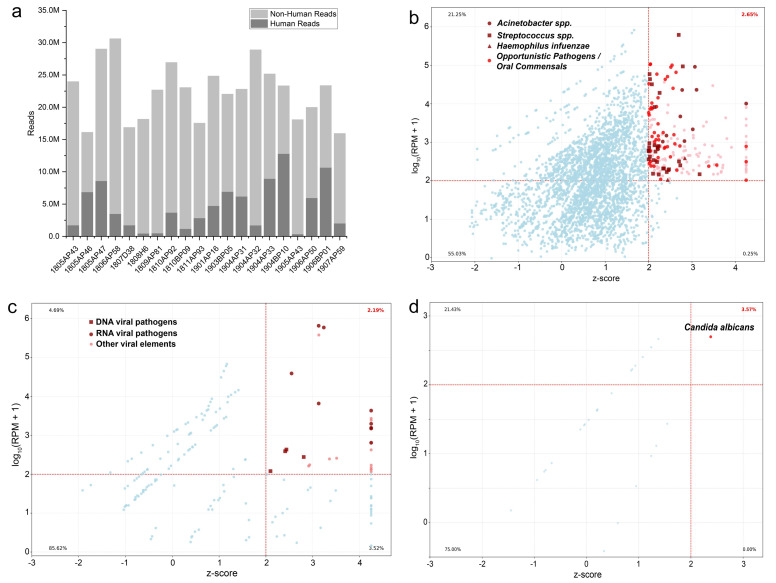
Overview of metatranscriptomic sequencing data and filtering strategy for pathogen identification. (A) Stacked bar chart showing the composition of sequencing reads across all 20 patient samples. Reads are categorized as Host-derived (human) or Non-human. (B–D) Scatter plots illustrating the distribution of all detected microbial taxa based on their abundance (expressed as log_10_(RPM + 1)) and statistical significance (Z-score) across all samples, separated by kingdom: (B) Bacteria, (C) Viruses, and (D) Fungi. The vertical dashed line indicates the Z-score threshold of 2, and the horizontal dashed line indicates the RPM threshold of 100. Taxa in the upper-right quadrant (shaded area) meet both criteria and were classified as significant potential pathogens for downstream analysis. The percentage shown in the top-right corner of each plot represents the proportion of reads from significant taxa relative to the total reads within each microbial kingdom. Selected clinically important pathogen taxa are differentiated by color and shape for emphasis.

A total of 20 inpatients diagnosed with severe pneumonia from various hospitals of Wuxi were included in the study, with ages ranging from 31–92, and male/female ratio 12:8. Deep sputum samples were collected from all patients, followed by RNA extraction to successfully construct transcriptome sequencing libraries based on the Illumina sequencing platform, which were then subjected to deep sequencing. The 20 libraries generated a total of 1.59  ×  10^7^ to 3.06  ×  10^7^ reads, with an average of 2.25  ×  10^7^. After quality control, on average, 32.63% of the sequencing reads were identified as host-derived (predominantly human), which were subsequently removed for downstream microbial analysis (Fig. 1A).

### Identification of significant pathogens

To distinguish potential pathogens from background microbiota and contaminants, we implemented a stringent bioinformatic and statistical filtering strategy. This dual-threshold approach effectively filtered out low-level, ubiquitous signals. As shown in Figs. 1B–1D, the vast majority of initially detected microbial reads fell below these thresholds, representing background or commensal flora. Specifically, only a small fraction of reads corresponding to bacteria (2.65%), viruses (2.19%), and fungi (3.57%) met our criteria for significance, allowing us to focus on microbes with high pathogenic potential in each patient.

**Figure 2:**
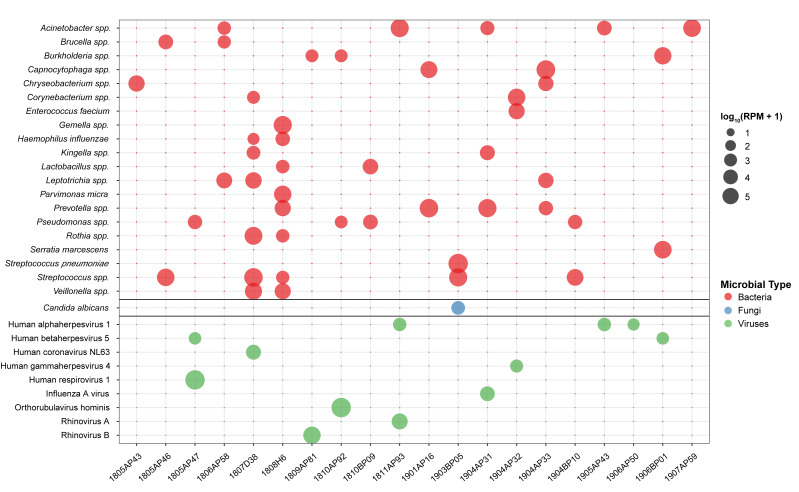
Distribution and abundance of significant potential pathogens across 20 severe CAP patients. This bubble heatmap displays all significant microbial taxa identified after applying stringent filtering criteria (RPM ≥ 100 and Z-score ≥ 2). Rows represent individual microbial taxa, and columns represent patient samples. Bubble color indicates microbial type (Bacteria, Fungi, or Viruses) as defined in the legend, while bubble size is proportional to microbial abundance, scaled as log_10_ (RPM + 1). Only taxa that met significance criteria in at least one sample are shown. For bacterial taxa, the taxonomic resolution varies depending on sequence homology: species-level identification (*e.g.*, *Haemophilus influenzae*, *Streptococcus pneumoniae*) was achieved when detected transcriptomic sequences were sufficiently unique to permit unambiguous assignment to a single species, whereas genus-level classification followed by “spp”. (*e.g.*, *Streptococcus* spp., *Prevotella* spp.) indicates that detected sequences share high similarity across multiple species within that genus, precluding definitive species-level assignment.

Following this filtering process, we characterized the retained high-confidence microbial signals. We identified diverse microbial communities with distinct potential pathogen profiles across the cohort. The microbial composition revealed substantial heterogeneity in both the types and abundance of detected organisms, with RPM values ranging from zero to over 600,000 for individual taxa (Fig. 2).

### Predominantly bacterial infections

Several samples exhibited profiles dominated by specific bacterial pathogens, suggestive of primary bacterial pneumonia or hospital-associated infections. The highest bacterial load in the dataset was identified in sample 1903BP05, which presented a distinct *S. pneumoniae* mono-infection at an exceptionally high abundance of 619,855 RPM. Notably, this sample also contained a trace amount of *Candida albicans* (498 RPM), the only fungal detection in the cohort, likely representing commensal background rather than active fungal disease.

Distinctive bacterial profiles involving organisms typically associated with healthcare settings or opportunistic infections were observed in other cases, despite sampling occurring at admission. Sample 1906BP01 showed a co-occurrence of *Serratia marcescens* (50,904 RPM) and *Burkholderia* species (33,055 RPM). Similarly, sample 1907AP59 was dominated by *Acinetobacter* species (58,247 RPM). Sample 1904AP32 displayed a unique profile characterized by a high abundance of *Capnocytophaga* species (223,187 RPM) alongside *Corynebacterium* species (41,985 RPM) and *Enterococcus faecium* (8,519 RPM). The detection of these pathogens at admission suggests probably healthcare-associated pneumonia (HCAP) risk factors of community-onset infections.

### Predominantly viral infections

Viral detection patterns were highly sample-specific, with several instances of high viral loads acting as the primary etiological agents. The highest overall microbial abundance in the study was attributed to *Orthorubulavirus hominis* in sample 1810AP92 (654,872 RPM). In this sample, bacteria such as *Pseudomonas* were detected only at negligible levels. Similarly, sample 1805AP47 was dominated by Human respirovirus 1 (596,391 RPM), accompanied by low levels of Human betaherpesvirus 5 (CMV) and *Pseudomonas* species, indicating an acute viral respiratory infection.

Rhinoviruses were identified as the primary drivers in two cases: sample 1809AP81 contained Rhinovirus B (38,942 RPM), while sample 1811AP93 showed Rhinovirus A (6,576 RPM) co-occurring with a low level of Human alphaherpesvirus 1 (HSV-1). HSV-1 was also sporadically detected in samples 1905AP43 and 1906AP50, likely reflecting viral reactivation or latent presence rather than primary infection.

### Polymicrobial and co-infection profiles

A subset of samples demonstrated complex community structures involving high-abundance co-occurrence of multiple bacterial species or bacterial-viral co-infections.

Sample 1807D38 presented a classic polymicrobial profile associated with aspiration. It contained a high load of *Streptococcus* species (170,822 RPM) co-occurring with *Rothia* (67,264 RPM), *Veillonella* (26,056 RPM), and *Leptotrichia* (10,777 RPM). Notably, Human coronavirus NL63 was also detected (1,980 RPM) in this sample, suggesting a bacterial-viral co-infection superimposed on an aspiration-type microbiome.

Sample 1808H6 exhibited a complex anaerobic community dominated by *Gemella* species (100,076 RPM), accompanied by significant levels of *Parvimonas micra* (36,483 RPM), *Prevotella* (8,709 RPM), and *Veillonella* (7,600 RPM). Haemophilus influenzae was also present at lower levels (1,197 RPM). This anaerobic-predominant pattern is strongly characteristic of aspiration pneumonia or lung abscess formation.

Sample 1901AP16 showed a high abundance of *Prevotella* species (168,067 RPM) and *Acinetobacter* species (99,123 RPM), alongside *Capnocytophaga* (19,760 RPM), indicating a mixed infection involving both oral anaerobes and potential nosocomial pathogens. Finally, sample 1904AP31 revealed a co-infection pattern with Influenza A virus (1,553 RPM) detected alongside a high burden of *Prevotella* species (105,811 RPM), pointing to a secondary bacterial infection following or concurrent with viral influenza.

### Identification and phylogenetic analysis of novel viruses

**Figure 3:**
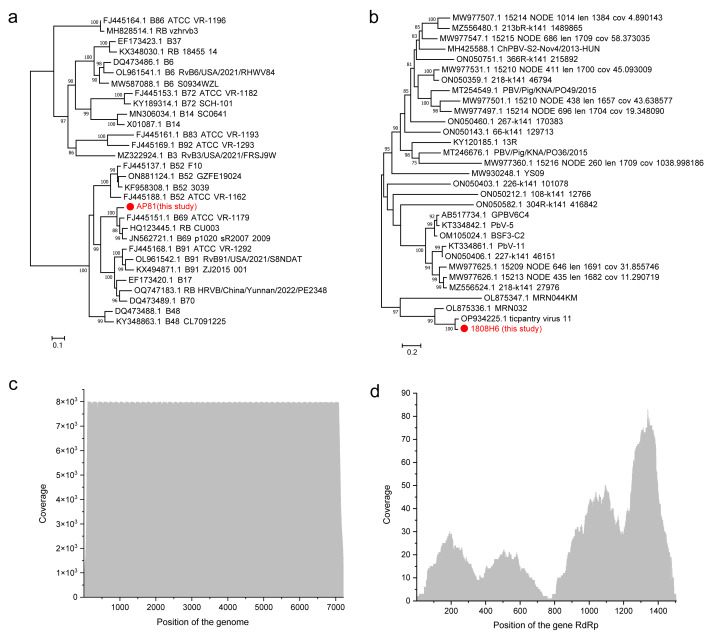
Characterization of two divergent viral strains identified in this study. (A, B) Maximum likelihood (ML) phylogenetic trees based on (A) complete or near-complete coding sequences of Rhinovirus B strains, including the novel strain AP81 identified in this study (highlighted), and (B) RdRp gene sequences of picobirnaviruses, including the novel strain detected in sample 1808H6 (highlighted). Bootstrap support values (based on 1,000 replicates) are shown at key nodes; only values ≥75% are displayed. Scale bars represent nucleotide substitutions per site. (C, D) Read coverage depth across the viral genome (or gene) for the novel strains: (D) coverage of the assembled Rhinovirus B AP81 genome and (D) coverage of the picobirnavirus RdRp gene fragment. The *x*-axis indicates genomic position (nucleotides), and the *y*-axis shows the number of mapped reads at each position.

Notably, a novel Rhinovirus B strain, hereafter designated AP81, was identified with high abundance in sample 1809AP81, representing the highest viral load among all detected viruses in this study (Figs. 3A and 3C). Phylogenetic analysis of its near-complete coding sequence revealed that AP81 forms a distinct lineage within the Rhinovirus B species (Fig. 3A). It clusters most closely with Rhinovirus B69 reference strains (*e.g.*, ATCC-VR-1179), forming a sister clade with strong bootstrap support (96%). Despite this grouping, the substantial branch length leading to AP81 visually represents significant genetic divergence, which is consistent with the calculated 90.52% nucleotide identity to its closest reference. This placement confirms it as a previously uncharacterized strain of Rhinovirus B.

Another novel viral strain, out of patient 1808H6, was belonging to *Picobirnaviridae*. Phylogenetic analysis based on the RNA-dependent RNA polymerase (RdRp) gene sequence places this virus in a well-supported clade (bootstrap value of 99%) with ticpantry virus 11, a recently discovered picobirnavirus from chimpanzee saliva (Fig. 3B). The RdRp gene of our strain shared 94.90% nucleotide identity with ticpantry virus 11. Importantly, this human/chimpanzee virus clade is clearly distinct from other known picobirnaviruses, including those from porcine sources (Porcine picobirnavirus) and other human respiratory samples previously classified as Orthopicobirnavirus hominis, with which it shares lower identity (83.48%–89.77%) (Dunay et al., 2023a; Dunay et al., 2023b). Despite its low abundance (Fig. 3D), the detection of this divergent picobirnavirus in a human respiratory sample is a notable finding, suggesting a potential zoonotic origin and expanding the known diversity of picobirnaviruses infecting humans.

## Discussion

We employed metatranscriptomics to comprehensively characterize the respiratory pathogen spectrum in sputum samples from 20 adult patients hospitalized with CAP in Wuxi, China, during 2018–2019. Our findings reveal the polymicrobial nature of CAP pathogenesis, with frequent co-detection of bacterial and viral pathogens. While comprehensive, the unbiased RNA-seq approach employed here involves complex library preparation and sophisticated bioinformatic analysis, making it relatively time-consuming and costly for routine clinical use. For clinical implementation, targeted and optimized mNGS/tNGS approaches—incorporating pathogen-specific enrichment or employing faster sequencing platforms with streamlined pipelines—may offer a more practical balance between turnaround time, cost, and diagnostic yield (Wu et al., 2020). Nevertheless, unbiased metatranscriptomics remains invaluable for etiological research. It provides a hypothesis-free view of active pathogens and host responses, which is critical for discovering novel or unexpected pathogens and understanding complex polymicrobial dynamics.

The bacterial pathogen landscape in our cohort was dominated by *Streptococcus* species, detected in five samples, with one case exhibiting monoinfection with exceptionally high *S. pneumoniae* abundance. This finding aligns with the well-established role of *Streptococcus* species in CAP etiology (Musher et al., 2020; Shoar & Musher, 2020). Notably, oral anaerobes such as *Prevotella*, *Veillonella*, and *Rothia*, along with certain *Streptococcus* species, were frequently detected at high abundance and transcriptional activity. Interpreting these findings requires distinguishing between genuine lower respiratory tract infection (*e.g.*, aspiration pneumonia) and oral contamination introduced during sample collection. While contamination typically presents as a diverse mixture of saliva-associated microbes, the quantitative profiles observed in specific samples suggest active infection rather than passive translocation. For instance, the exceptionally high microbial loads and the distinct dominance of specific anaerobic taxa over a background of diverse oral flora are consistent with the outgrowth of aspirated flora in the lower airways—a hallmark of anaerobic aspiration pneumonia or lung abscess (Bartlett, 1993; Dickson et al., 2016). Nevertheless, we acknowledge that without quantitative culture validation or concurrent oral sampling, the contribution of oral contamination cannot be entirely ruled out. Therefore, integration with clinical parameters—including radiological findings (*e.g.*, infiltrate distribution suggestive of aspiration), inflammatory markers, and response to antimicrobial therapy—is essential to definitively determine the pathogenic significance of these organisms.

*Haemophilus influenzae*, a well-documented CAP pathogen particularly in patients with chronic obstructive pulmonary disease (Zhang et al., 2019), was detected in two samples, though at relatively low abundances. This low detection rate may reflect the study population characteristics, pre-existing antibiotic exposure, or temporal variation in pathogen circulation. The absence of *M. pneumoniae*, a common cause of CAP especially in younger populations and outbreaks (Kutty et al., 2019), across all samples is noteworthy. This may reflect the age distribution of our cohort (hospitalized adults), the severity-based selection (*M. pneumoniae* typically causes milder disease), or the epidemiological context of the 2018–2019 period in Wuxi. Indeed, *M. pneumoniae* exhibits cyclical epidemic patterns, and our study period may have coincided with an inter-epidemic interval.

Viral pathogens were detected in five samples, with several cases demonstrating high viral loads indicative of acute viral pneumonia. *Orthorubulavirus hominis* and Human respirovirus 1 exhibited the highest viral abundances, suggesting these were likely the primary etiological agents in these cases. Herpesviruses were detected in four samples (20%), including CMV, HSV-1, and Human gammaherpesvirus 4 (EBV). These detections likely represent viral reactivation in the context of severe illness and immunosuppression rather than primary infections. The transcriptional activity of these typically latent viruses underscores the immunological stress and microbiome dysbiosis experienced by severely ill CAP patients (Liu et al., 2023b; Meyer et al., 2021). However, their contribution to disease pathogenesis *versus* representing bystander reactivation remains uncertain.

Our identification of a divergent Rhinovirus B strain AP81 with high abundance in sample 1809AP81 is noteworthy. We assembled a near-complete coding sequence showing only 90.52% nucleotide identity to its closest reference (RhV-B69), indicating substantial genetic divergence. While rhinovirus infections typically cause mild upper respiratory symptoms, certain strains have been associated with severe lower respiratory tract infections, particularly in adults with comorbidities (Morelli et al., 2025). The clinical significance and virulence determinants of strain AP81 warrant further investigation, as does epidemiological surveillance for its circulation.

The detection of a novel picobirnavirus strain phylogenetically related to ticpantry virus 11 from chimpanzee saliva raises questions about potential zoonotic origins (Dunay et al., 2023b). Picobirnaviruses have been detected in various mammalian hosts, and phylogenetic evidence suggests capacity for cross-species transmission (Sadiq, Holmes & Mahar, 2024). However, interpretation of this specific finding requires caution due to low viral abundance (RPM < 100) and limited genome coverage. The uneven coverage pattern likely reflects technical limitations associated with low viral titers. Nevertheless, this detection, while likely not clinically significant in this case due to low abundance, serves as a proof-of-principle that metatranscriptomics can identify potential spillover events or novel pathogens that would be missed by culture or targeted polymerase chain reaction (PCR)-based diagnostics.

Several important limitations should be acknowledged. First, the absence of sequenced negative controls (*e.g.*, no-template controls) is a notable limitation. To address this, we implemented a rigorous computational decontamination strategy combining Kraken2/Bracken classification with stringent statistical filtering. While this approach effectively removed ubiquitous, low-abundance contaminant signals, future studies should incorporate sequenced negative controls to provide additional confidence. Second, RNA-seq is biased toward detecting RNA viruses and transcriptionally active microorganisms. DNA viruses, such as herpesviruses, may be underrepresented if in latent states with low transcriptional activity. Third, the sample size was relatively small (*n* = 20) and geographically limited, which may affect generalizability regarding pathogen prevalence. Regional and temporal variations in CAP etiology are well-documented, and our findings represent the pre-pandemic period (2018–2019) in Wuxi specifically. Fourth, metatranscriptomic pathogen detection does not inherently establish causality. While our filtering strategy prioritizes likely pathogens, clinical correlation is necessary to determine true pathogenic significance. We lacked detailed clinical metadata including disease severity scores, antibiotic exposure history, comorbidities, and outcomes, which would strengthen causal inference. Additionally, we did not compare our findings with conventional diagnostics (culture, PCR) for the same patients, which would be valuable validation. Fifth, our single-timepoint sampling strategy (at admission) provides only a snapshot. Longitudinal sampling could reveal pathogen dynamics, secondary infections, or viral reactivation during hospitalization.

Some research priorities emerge from present study. First, larger multicenter studies with geographically diverse populations are needed to validate our findings and establish comprehensive baseline data on adult CAP etiology in China. Second, longitudinal surveillance tracking temporal and seasonal variations would provide critical context for interpreting epidemic patterns, particularly post-COVID-19. Third, functional studies investigating virulence mechanisms of novel strains (*e.g.*, Rhinovirus B strain AP81) would clarify their pathogenic potential. Fourth, integration of metatranscriptomic data with detailed clinical parameters, treatment regimens, and outcomes could establish clinical significance of detected pathogens and guide evidence-based treatment strategies. Fifth, comparative studies incorporating both RNA-seq and DNA-seq would provide a more complete pathogen landscape, including latent DNA viruses. Finally, direct comparison with conventional diagnostics in prospectively enrolled cohorts would validate the clinical utility of metatranscriptomics.

## Conclusions

Our metatranscriptomic analysis of 20 adult CAP cases in Wuxi (2018–2019) revealed a diverse pathogen landscape, dominated by *Streptococcus* species and oral anaerobes, and characterized by frequent polymicrobial infections. We identified significant viral pathogens including *Orthorubulavirus hominis* and Human respirovirus 1, and characterized two divergent viral strains: a novel Rhinovirus B strain and a picobirnavirus related to simian viruses. This study provides pre-pandemic baseline data for adult CAP etiology in Wuxi and enables future assessments of post-pandemic epidemiological shifts. Metatranscriptomics is an invaluable research tool for pathogen discovery, surveillance, and understanding infection complexity. Our findings underscore the polymicrobial nature of CAP and highlight the need for comprehensive diagnostic approaches capable of detecting diverse pathogen types, ultimately informing evidence-based treatment strategies and improving patient outcomes.

##  Supplemental Information

10.7717/peerj.20774/supp-1 Supplemental Information 1 Code used for data analysis The code package consists of three main components:
Main Analysis Pipeline: Covering data preprocessing, reads assembly, Diamond BLASTX alignment, and quantification.
R Scripts: Used for integrating contig quantification with taxonomy information.
Kraken2/Bracken Scripts: Used for read-based quantification and analysis.

10.7717/peerj.20774/supp-2 Supplemental Information 2 Inclusion and exclusion criteria of the patients enrolled

## Additional Information and Declarations

### Data Availability

The following information was supplied regarding data availability: The code is available in the Supplemental File. The gene sequences are available at NCBI Genbank: OR735056 ∼OR735057. The raw sequence data are available at CNGB (China National GeneBank DataBase): CNP0006897; CNX1182905–CNX1182924.
