# Supplementary material for: A retrospective metatranscriptomic study of respiratory pathogens causing adult community-acquired pneumonia in Wuxi, China, before the pandemic
Source: PeerJ. 2026 Feb 10;14:e20774. doi: 10.7717/peerj.20774 (PMC12903900; doi:10.7717/peerj.20774)
Supplement: Supplemental Information 2 [file peerj-14-20774-s002.docx]

The following criteria were established based on the "Diagnosis and treatment of community-acquired pneumonia in adults: 2016 clinical practice guidelines by the Chinese Thoracic Society" (Cao et al., 2018) and specific requirements of the study protocol.

**Inclusion Criteria**

Patients must meet all of the following criteria to be included in the study:

1. Age:

Patients aged 18 years or older.

1. Clinical and Radiological Diagnosis of Community-Acquired Pneumonia (CAP):

A diagnosis of CAP established according to the 2016 CTS guidelines, which requires the fulfillment of criteria A, C, and at least one item from B, after excluding other relevant diseases.

A. Onset in the community.

B. Relevant clinical manifestations of pneumonia (at least one of the following):

1. New onset of cough or expectoration, or aggravation of existing respiratory symptoms, with or without purulent sputum, chest pain, dyspnea, or hemoptysis.
2. Fever.
3. Signs of pulmonary consolidation and/or moist rales on auscultation.
4. Peripheral white blood cell count (WBC) > 10 × 10⁹/L or < 4 × 10⁹/L, with or without a left shift.

C. Radiological Evidence:

Chest radiograph showing new patchy infiltrates, lobar or segmental consolidation, ground-glass opacities, or interstitial changes, with or without pleural effusion.

**Exclusion Criteria**

Patients meeting any of the following criteria were excluded from the study:

1. Non-Community-Acquired Pneumonia:

Pneumonia acquired in a hospital setting (Hospital-Acquired Pneumonia).

1. Age:

Patients younger than 18 years.

1. Severe Immunosuppression:

Patients considered to be immunocompromised as defined by the guideline, including but not limited to those with:

1. Human immunodeficiency virus (HIV) infection.
2. Agranulocytosis.
3. Haematological tumour or solid tumour undergoing chemo-radiotherapy.
4. Solid organ transplantation.
5. Receiving long-term glucocorticoid or cytokine antagonist therapy.
6. Alternative Diagnoses:

Patients for whom a final diagnosis other than CAP was made. The guideline lists the following to be excluded for a CAP diagnosis:

1. Tuberculosis
2. Pulmonary tumour
3. Non-infectious interstitial lung disease
4. Pulmonary edema
5. Atelectasis
6. Pulmonary embolism
7. Pulmonary eosinophilia
8. Pulmonary vasculitis
9. Study-Specific Exclusions:

The following criteria were applied based on the specific requirements of the study protocol:

1. Prior Antibiotic Use: Use of systemic antibiotics within 48 hours prior to hospital admission.
2. Sputum Sample Unavailability: Inability to produce a sputum sample or providing a sample of inadequate quality for microbiological analysis (e.g., squamous epithelial cells > 10 per low-power field).
